# An Overview of the Use of Nanoparticles in Vaccine Development

**DOI:** 10.3390/nano13121828

**Published:** 2023-06-09

**Authors:** Daniel Lozano, Vicente Larraga, María Vallet-Regí, Miguel Manzano

**Affiliations:** 1 Departamento de Química en Ciencias Farmacéuticas, Instituto de Investigación Sanitaria Hospital 12 de Octubre i + 12, Universidad Complutense de Madrid, Plaza Ramón y Cajal s/n, 28040 Madrid, Spain; danlozan@ucm.es; 2 Networking Research Center on Bioengineering, Biomaterials and Nanomedicine (CIBER-BBN), 28029 Madrid, Spain; 3 Laboratorio de Parasitología Molecular, Unidad de Desarrollo de Fármacos Biológicos, Inmunológicos y Químicos para la Salud Global (BICS), Departamento de Biología Celular y Molecular, Centro de Investigaciones Biológicas Margarita Salas, Consejo Superior de Investigaciones Científicas (CIBMS-CSIC), 28040 Madrid, Spain; vlarraga@cib.csic.es

**Keywords:** vaccine, mRNA, DNA, nanoparticles, lipids, SARS-CoV-2, biomedicine, therapy, biotechnology

## Abstract

Vaccines represent one of the most significant advancements in public health since they prevented morbidity and mortality in millions of people every year. Conventionally, vaccine technology focused on either live attenuated or inactivated vaccines. However, the application of nanotechnology to vaccine development revolutionized the field. Nanoparticles emerged in both academia and the pharmaceutical industry as promising vectors to develop future vaccines. Regardless of the striking development of nanoparticles vaccines research and the variety of conceptually and structurally different formulations proposed, only a few of them advanced to clinical investigation and usage in the clinic so far. This review covered some of the most important developments of nanotechnology applied to vaccine technologies in the last few years, focusing on the successful race for the preparation of lipid nanoparticles employed in the successful anti-SARS-CoV-2 vaccines.

## 1. Introduction

In a very broad definition, vaccination consists of the delivery of certain agents, so-called antigens, into the body to trigger the immune response and generate adaptive immunity against certain pathogens [1]. Generally speaking, vaccination is a simple, safe, and effective method to protect people against harmful diseases. The process involves the body’s natural defenses to build resistance to some specific infection making the immune system stronger. Along the years, it was demonstrated that vaccination is the most efficient way of preventing and even eradicating several infectious diseases, such as smallpox, polio, or diphtheria, among others [2].

The development of vaccines throughout the 20th century should be contextualized, as it was one of the greatest medical successes in disease control and prevention [3]. In fact, the average life expectancy increased by more than 40 years in just one century [4], mainly thanks to three basic technological advances, most of them related to the improvement of living conditions for children: (1) improved sanitation and channeling of water and sewage, which prevented many diseases; (2) the development of antibiotics to fight infections; and (3) the development of vaccines to prevent a large number of diseases. 

Initially, the development of vaccines was based on the paradigm described by Louis Pasteur, which included isolating, inactivating, and injecting the disease-causing pathogen. In fact, as commented below, vaccines were based on the use of either live attenuated pathogens [4] or inactivated pathogens [5] or their subunits that trigger a particular immunological activation. However, there are still many infectious diseases that cannot be prevented with any vaccine. This failure is normally a consequence of the inability to evoke appropriate immune responses [6]. 

There are different vaccine administration routes [7], although most of them are commonly administered via intramuscular or subcutaneous routes, with others being administered intradermal or orally [8]. However, many conventional vaccines, independently of their administration route, present some issues, such as instability or toxicity. The application of nanotechnology to vaccine development promised many advantages, such as better lymph node accumulation, antigen assembly and presentation, and unique pathogen biomimicry properties [9].

Nanoparticles are a broad class of materials with a dimension of less than 100 nm. Their unique physical and chemical properties, together with their size, shape, and structure, fueled their applications in many different areas, such as catalysis, imaging, energy, environmental, or even biomedical, applications. In this sense, a potential alternative to traditional vaccines could be using nanoparticles able to display or transport antigens [10,11]. The successful clinical application of nanoparticles-based Moderna and Pfizer/BioNTech COVID-19 vaccines highlighted the promising future of the application of nanotechnology to vaccine development. 

In this review, we described some of the latest vaccine technologies, including mRNA and DNA technologies, and the different approaches of nanotechnology to vaccine development. The different nanoparticles employed as vaccines were described together with their interaction with the body, and a success case of vaccines based on nanoparticles was deeply reviewed. 

## 2. Conventional Vaccines

As stated by the WHO, vaccines are pharmaceutical formulations that generate protective immunity against a disease by activating the production of specific antibodies by the immune system against a pathogen [12]. Vaccines are considered the most effective method to control epidemics, such as measles, diphtheria, and, eventually, SARS-CoV-2. The first vaccine used in the Western world was discovered by E. Jenner at the end of the eighteenth century against smallpox, a terrible cause of mortality at the time [13]. This vaccine was based on the cowpox virus. E. Jenner was a rural physician who realized that cattle workers who recovered from cowpox disease were protected against smallpox. However, he did not know that the disease was caused by a virus; an infectious agent that was not discovered and described until the twentieth century. 

From a scientific point of view, classical vaccines are a direct consequence of the discovery by E. von Behring and S. Kitasato, at the end of the nineteenth century, of the presence of protective molecules, coined antibodies, in the plasma of patients who recovered from infections caused by bacteria, virus, or fungi [14]. These molecules also protected patients from future attacks by the same pathogen. This discovery generated a rush among microbiologists to identify the pathogens responsible for common infectious diseases at the time, including diphtheria, measles, and poliomyelitis, etc., to subsequently obtain protective vaccines. Antigens used to acquire protection include attenuated pathogens which lost the ability to infect after repetitive cultures in the laboratory (attenuated vaccines), such as the Calmette–Guerin vaccine based on *M. bovis*, against tuberculosis, and pathogens inactivated by heat or chemical agents (inactivated vaccines) [14,15]. In many cases, functional antigens for vaccines were composed of purified fragments of pathogens that elicited an immune response against the entire bacteria or virus. 

Conventionally, these antigens were obtained by DNA recombinant processes in other organisms, mainly bacteria or culture cells that produce high quantities of the selected antigen from its encoding gene, which can be easily purified [16]. These biological preparations used in vaccinations were called second generation vaccines. This generation is based on subunit elements, recombinant or synthetic proteins, non-protein antigens, and expressed bacterial or viral immunogens, which may include numerous molecules and epitopes of different strains or even species of pathogens [17].

A new method was developed in the last few years. The mechanism used to introduce protective antigens belonging to distinct pathogens to elicit a specific immune response and to subsequently confer protection against the disease took a step further [14,18]. This new method avoids the long and costly process of antigen purification and increases the effectivity. This novelty consists of introducing each protective antigen’s encoding gene into a mammal, leading to its internal production and recognition by the host’s immune system, and subsequently changing the internal mechanism of processing it [19]. This method improves antigen processing and presentation to antigen presenting cells (APCs) and activates the CD4^+^ and CD8^+^ cells more effectively than introducing the protective antigen, purified by an external procedure [18]. The use of both DNA and RNA molecules was introduced, resulting in a qualitative advance in eliciting specific protective immune responses [20,21]. These are known as third generation vaccines. These vaccines are based on introducing a gene encoding a pre-selected protective antigen into host cells using a vaccine vehicle. The vehicle can be a recombinant one, usually a virus, such as the adenovirus responsible for the chimpanzee cold, which was modified by incorporating a pathogen’s antigen-encoding gene in its genome. Host cells are infected by the virus and the protective antigen is produced by the cells and recognized as foreign by the host’s immune system, inducing a specific response in a more efficient process. Although this method is effective, several inconveniences exist. The vaccine antigen is one of the specific responses elicited together with the rest of viral proteins, which somehow dilutes the intensity of the immune response and reduces the effectivity of the second dose of the vaccine as the vehicle is also recognized as foreign by the host’s immune system [22]. 

## 3. Nanotechnology in Vaccines

In the last few decades, nanotechnology was applied on a variety of scientific fields, including medicine, which gave rise to the birth of a new scientific discipline called nanomedicine [23]. This relatively recent field became a multidisciplinary scientific area with many researchers involved, such as engineers, physicists, chemists, biologists, physicians, and even legislators [24]. One of the benefits of nanomedicine is its nanometric scale, which is the scale of many biological mechanisms in the human body [25]. This fact allows many nanoplatforms to cross some natural barriers and, therefore, access new sites of delivery and/or interact with DNA or proteins at different levels, in different organs, tissues, or cells. Nanomedicine is expected to be a very important instrument for personalized, targeted, and regenerative medicine thanks to the development of new treatments that could be breakthroughs in healthcare [26]. 

### 3.1. New Vaccine Technologies

Over the years, conventional vaccines saved lives and prevented disease, eradicating smallpox and reducing the incidence of other diseases such as polio and measles. The next generation of vaccines focused on obtaining similar efficacy to conventional vaccines, but without their risks or limitations. This was achieved through improved knowledge in areas such as immunology, pathology, and microbiology, which helped to adopt more rational designs. These minimalist compositions provide improvements in safety and production costs, although they may lead to lower immunogenicity [27]. 

Although vaccines with attenuated or inactivated viruses improved the quality of life and reduced mortality, they still have a number of shortcomings, such as less than optimal reactivity or efficacy, and are still very expensive and time-consuming to produce [28]. In addition, this conventional technology was not able to develop vaccines against a number of contemporary diseases. These drawbacks, coupled with the fear of the emergence of new infectious agents that could lead to a pandemic, led to research into other possible options, including RNA or DNA vaccines [21,28].

#### 3.1.1. mRNA Technology

**Figure 1:**
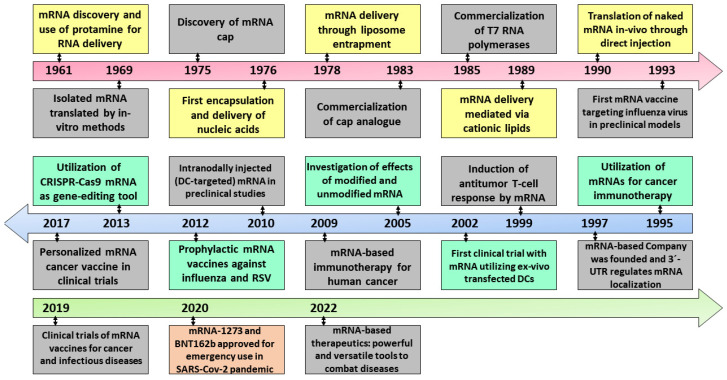
Historical evolution of the different stages of RNA development and its therapeutic applications from the mRNA discovery in 1961 to the development of mRNA therapies in 2022. Reproduced from [29].

The rapid design and development of the COVID-19 mRNA vaccines marked the beginning of a new biotechnology platform that was not only useful for immunization against SARS-CoV-2 but can potentially be applied to a broad spectrum of microbial pathogens and cancers. The short timelines and safety profile obtained with mRNA vaccines tested in millions of humans suggest the efficacy of these systems as vaccines [29]. Although the study and production of mRNA vaccines took place in record time, this would not have been possible without decades of study and work on the molecular and cellular biology of mRNA and its possible applications (Figure 1) [30,31].

**Figure 2:**
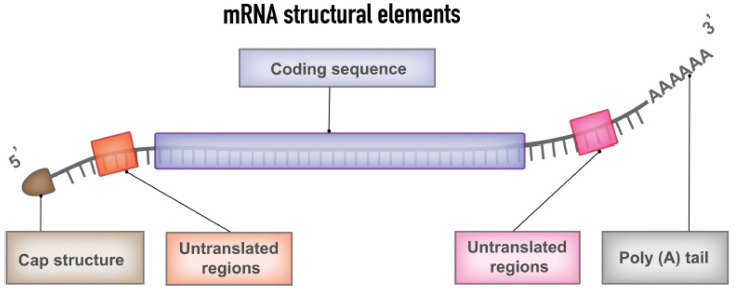
Representation of the different mRNA structural elements that are included in mRNA vaccine, including the cap and tale fragments, the untranslated regions, and the coding sequence. Reprinted with permission from [32] 2022, Frontiers.

Messenger RNA is a genetic molecule that plays a crucial role in the protein manufacturing process in our body. The central dogma of molecular biology begins with the information contained in the DNA, which is stored in the nucleus of each of our cells. DNA is converted, inside the cells, into messenger RNA that is responsible for transporting the information necessary for the manufacture of proteins, which are required for many our body’s functions. The typical mRNA consists of a cap flanked by 5′-untranslated regions (UTR), 3′-UTR, an open reading frame encoding antigens and a poly(A) tail (Figure 2). The coding region, non-coding region, and optimized delivery formats can be altered to increase efficacy, stability, and immunostimulatory properties [32,33].

There are many diseases that are usually related to some error in the functioning of certain proteins, either because there is too much of a certain protein, or too little, or a mutation that causes it not to have the correct form or not to function as it should [34]. This inspired the idea of the possibility of treating those diseases with messenger RNA technology. 

What really empowered RNA as a therapeutic agent is its role in the protein production chain. The fact that it is a temporary intermediate allowed its modification to explore protein synthesis without affecting the DNA permanently [35]. In this sense, it is possible to manufacture any protein, even a protein that our body cannot manufacture by itself, by designing a messenger RNA sequence and introducing it into the body. Therefore, the body is responsible for the synthesis of that protein. In this way, we can induce the expression of a large number of proteins that could potentially help in several genetic treatments or, in the case of SARS-CoV-2 vaccines, prevent diseases [35].

However, the possibility of genomic integration of therapeutic synthetic mRNA should not be discarded, as it can occur—with very low frequency—with plasmid DNA-based vaccines or some vectorized vaccines [36,37]. In this sense, it could be possible that a nucleoside-modified synthetic mRNA could activate the expression of some internal transposable elements and undergo reverse transcription entering the nucleus of the cell [38].

In any case, mRNA technology has great potential for various genetic treatments, such as therapies based on protein replacement, as in the case of hemophilia, where patients lack a particular protein in the blood that helps blood clotting. If the mRNA sequence of that protein is developed and administered, the body itself will be able to synthesize that protein and, thus, enable the blood to clot. Messenger RNA can also be used in cancer immunotherapy, from the point of view of designing mRNA encoding proteins that can teach our immune system how to attack tumor cells in a specific way [34,35]. Messenger RNA is also being investigated to create the famous transcription factors, which are a type of proteins that condition our body’s stem cells differentiate into certain cells, so we can help our stem cells to create new types of tissues. It also has applications in gene editing by silencing the expression of certain proteins. Additionally, of course, in the development of vaccines, that is the most advanced clinical application, in which mRNA is introduced so that our body can synthesize an antigen protein. In the case of SARS-CoV-2, we introduce messenger RNA that codes for small proteins that are normally found on the surface of the virus, so the immune system learns that this protein may be a potential enemy and it will fight it the next time it encounters it.

The development of mRNA therapies presents major complications than those found for mRNA vaccines. For example, immunization requires a minimal amount of protein production, whereas mRNA therapies require 50–1000 times more protein to be effective, as demonstrated in different in vivo mouse models in oncology [29,32,39]. To address this problem, efforts are being made to optimize mRNA loading in order to minimize innate immune responses and improve mRNA stability. However, mRNA cargo properties must be considered in relation to the efficacy of the delivery system and the mode of action of the protein of interest. Hence, targeting and uptake by the target tissue may be more important than other factors, with effective delivery to solid organs being a challenge. In addition, in many chronic diseases, multiple doses are required throughout disease progression, which may attenuate the expression of the therapeutic protein through activation of innate immunity [32,34]. 

#### 3.1.2. mRNA Delivery Platforms

The real problem of mRNA as a therapeutic agent is that it cannot be administered orally or intravenously, since it would be degraded by our body’s nucleases and would activate the immune system [29,32,34,39]. The original function of mRNA is to transport genetic information from the nucleus to the ribosomes where proteins are produced, but always inside the cells and never outside them. Our body has a large number of nucleases prepared to fight against any RNA or DNA that is circulating in the bloodstream, as it could be any pathogen that could cause us any disease. This is why naked mRNA cannot be administered and this is where drug delivery. We technology, and more specifically nanoparticles, helped to encapsulate mRNA to protect and transport it to take this technology to the next level [29,39].

##### Lipid Nanoparticles

**Figure 3:**
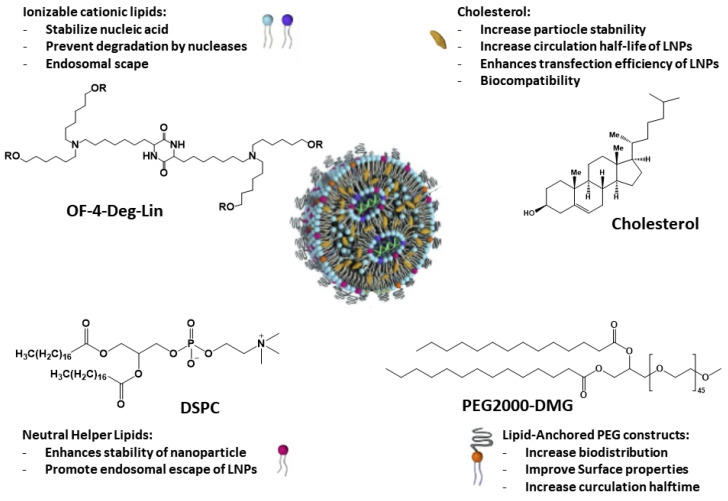
Description of the mRNA-based lipid nanoparticle general structure and their basic components together with their main characteristics. Reproduced from [41,42].

Due to the emergence of SARS-CoV-2, two mRNA-based vaccines developed using lipid nanoparticles (LNPs) received emergency use authorization from the US FDA for clinical use (Moderna and Pfizer/BioNTech) (Figure 3) [40,41,42]. This overcame initial skepticism about the use of biotechnological approaches based on nanoparticles. Especially when nanoparticles have unique advantages such as protection of cargo from degradation (very important for mRNA, as stated above), increased surface area and modulation of drug pharmacokinetics.

In this regard, early data revealed that BNT162b2 and mRNA-1273 have an efficacy of 95% and 94.5% against SARS-CoV-2, respectively. The Moderna vaccine is based on a stabilized mRNA of the viral spike protein, and BNT162b2 on a nucleoside-modified RNA (modRNA) of the SARS-CoV-2 virus [40]. Although in the case of SARS-CoV-2, other vaccines were developed based on other approaches, mRNA-based vaccines received priority clinical approval, as the technology ensures the mRNA stability, along with greater mRNA transport and delivery efficiency into the host cell. As non-infectious vaccines, they are safer and do not require penetration of the cell nucleus and can be produced rapidly. On the other hand, mRNA transport, conservation, and cellular internalization are complicated by the enzymes that can degrade it and the negative charge of the cell membrane. These two facts were neutralized by the design of LNP-based carrier molecules to preserve the integrity of the mRNA and favor its internalization within the cell [40]. LNPs are an FDA-approved carriers that are widely used to carry mRNA encoding antigens, encapsulating viral antigens against cytomegalovirus, human immunodeficiency virus, influenza and rabies, among others [43].

Liposomes were employed as drug delivery systems since the 1960s–70s and were sophisticated over time for disease-targeted delivery [39,40,44]. Liposomes are small spherical vesicles formed by one lipid bilayer (unilamellar liposomes) or several lipid bilayers (multilamellar liposomes). Lipid nanoparticles, unlike classical liposomes, form micellar structures within the nucleus. The structure of LNPs consists of a central solid lipid core, which is composed of triglycerides or glycerides. As can be observed in Figure 3, LNPs consist of four parts (a) an ionizable lipid portion that allows self-assembly, increases the speed of mRNA encapsulation, and aids endosomal escape, (b) cholesterol or a sphingolipid as a stabilizing agent for membrane stability and fusion, (c) a phospholipid that stabilizes the bilayer, encapsulating the lipid structure, and (d) a lipid-based stabilizing agent (polyethylene glycol-conjugated lipids), which increases the half-life, increases circulation time, and reduces non-specific binding to proteins [39,40,44]. Possessing rigid morphology, kinetic stability, low cytotoxicity, and immunogenicity make LNPs effective transporters of a diverse group of drugs, including nucleic acids, since ionizable lipids have a near-neutral charge at physiological pH, but in acidic endosomes, they ionize, favoring the endosomal escape and, therefore, mRNA release inside cells [45].

As previously discussed, labile mRNA requires an encapsulation system to protect it from degradation by nucleases and to allow cellular internalization, releasing its contents into cells and inducing translation into proteins. Although most mRNA therapeutics are linked to LNPs, preclinical studies are underway with other encapsulation systems, such as cells, extracellular vesicles, and biomimetic vesicles [32,39]. 

##### Human Cells

Mesenchymal, blood, or immune cells were used as transporters of different types of drugs to other target cells. An alternative to the use of LNPs is to use cellular paracrine function to directly transport mRNA introduced into cells ex vivo to target cells by genetic engineering. This system provides increased endogenous intercellular signaling, enhanced biocompatibility and longer duration in the bloodstream, but also has limitations in its application due to different national legislations, donor compatibility, and homogeneous production.

##### Extracellular Vesicles

Another approach currently being studied is the use of extracellular vesicles (EVs) as vehicles [46]. Evs are a heterogeneous group of extracellular bilayers produced by almost all cell types, including exosomes (50–150 nm), which are processed by the endosome as well as LNPs. These Evs also have good biocompatibility and immunogenicity and are being used in diagnosis, prognosis, and therapeutics of different diseases. In this regard, the cellular source from which these EVs are derived is crucial, as it was shown that EVs can reproduce the properties of the cells from which they originate and could also work for their specific delivery, in combination to their transport function. The limitations associated with this approach focus on the characterization, isolation, and purification of homogeneous EVs as well as the loading and release of the drugs that they carry [46]. 

##### Biomimetic Nanoparticles

On the other hand, the combination of biological and synthetic particles (biomimetic encapsulation), using a synthetic core (LNPS, silica) and a membrane coating of different cell types such as mesenchymal, immune, or tumor cells, or fusing both components, is still under preliminary study. The cell membranes stabilize the particle in the bloodstream while targeting and decreasing the immunogenicity of the synthetic part. The advantages of these systems combine the individual benefits from both components, i.e., they are biocompatible and specific and are capable of being manufactured and stored in a stable manner with a great drug loading capacity [32,39].

Based on what was explained in this section and taking into account its application in the case of SARS-CoV-2, it seems that mRNA is a good candidate for different therapeutic approaches. This path did not begin in 2019 but came from the effort and work of dozens of years of scientific and clinical advances. Thanks to all that research, it was possible to manufacture the mRNA in a scalable and massive way, encapsulate it, and release it efficiently. Overcoming the current limitation of being transported, keeping the cold chain and its possible freeze-drying would solve distribution problems throughout the world, not only for SARS-CoV-2 or other viral infections, but also for its application in a wide range of diseases [47].

#### 3.1.3. DNA Technology

Nucleic acid vaccines, including DNA vaccines for animal and human diseases as well as RNA vaccines for cancer and viral infections, were studied in the last twenty years to improve recombinant vaccines and reduce their limitations [21,48,49]. mRNA vaccines were described in other part of this review and will not be considered here. In this sense, DNA-based vaccines were more actively developed than mRNA-based vaccines during the last few years.

The initial step for the development of DNA vaccines is the procurement of a transport vehicle for delivery of the encoding gene of the selected protective antigen into vaccinated host’s cells. The gene is introduced into a polylinker region that contains the synthetic plasmid. The transfected cells will produce the selected protein and secrete it to the extracellular media. This protein, recognized as foreign, will elicit a protective response of the host immune system. These plasmids are grown in *E. coli* strains for vaccine production and must be selected from the total bacterial population. This was usually carried out by introducing genes of antibiotic resistance in the whole sequence and will grow in media containing the selected antibiotic, usually neomycin or kanamycin [50], which is adequate for laboratory developments. However, most of the regulatory agencies do not accept vaccines that might contain an antibiotic resistance gene. Then, plasmids with alternative selection genes were introduced in recent years to obtain safer vaccines [51]. As an additional precaution, plasmids containing the encoding genes of the protective antigens are designed for having just one round of replication [52]. This avoids the possibility of integration of the selected gene into the genome of the vaccinated mammalian host.

**Figure 4:**
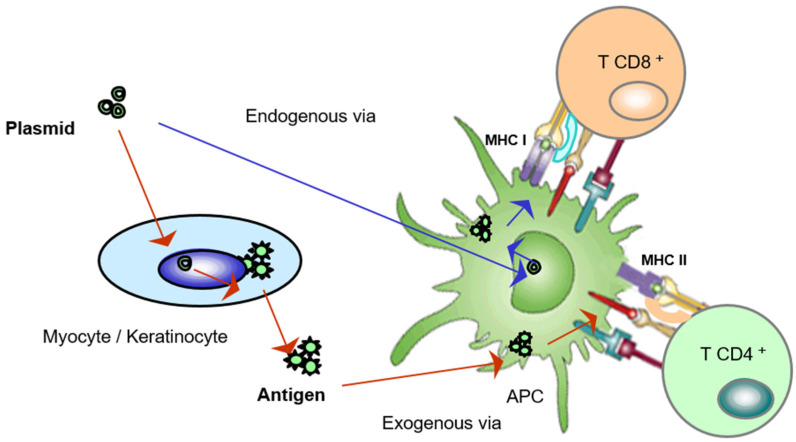
Mechanisms of activation of an antigen presenting cell through a DNA vaccine. This activation can be either directly (endogenous via) or through an intermediate cell that captures the plasmid and produces the pathogen antigen that will be recognized by the APC (exogenous via).

The activation mechanism for eliciting a protective reaction by the immune system of the host through a DNA vaccine is shown in Figure 4. DNA vaccines, which are synthetic DNA plasmids containing the appropriate signals to enter host cells, mainly keratinocytes and myocytes, induce in the nuclei of the patient’s cells one round of replication to produce the specific mRNA. This mRNA will be then sent to the cytoplasmic zone of ribosomal complexes to be used to synthesize the selected protein antigen in the cytoplasm [53]. The antigen is then secreted from the cell and detected and phagocytosed by an antigen presenting cell (APC), which will activate CD4^+^ cells, in the so-called exogenous via. These activated CD4^+^ cells trigger B cell proliferation and the production of specific antibodies that block the antigen and the pathogen in the case of infection. Additionally, T and B memory cells are produced to protect from future infections. Alternatively, the inoculated DNA plasmid can be captured directly by an APC. In this case, (endogenous via) once the antigen is synthesized, it is presented by an internal pathway to CD8^+^ cells, generating cytotoxic lymphocytes that eliminate infected host cells, completing the protective action against pathogen infection [54]. In the case of RNA molecules, which are now being successfully used against viral epidemics, the mechanism of action is similar, except for the initial mRNA synthesis in the nucleus, which is not necessary. mRNA penetrates this key cell in the immune response. On the contrary to DNA, RNA is very easily degraded by temperature variations and has to be stabilized using lipid particles, as was mentioned above [55].

DNA vaccines were mainly studied and developed for animal health diseases. A DNA vaccine against haemorrhagic fever in salmonids was available for almost five years [48]. This type of vaccine is being used against SARS-CoV-2 infection in India [56]. Recently, a DNA vaccine against canine leishmaniasis was accepted by the European Medicines Agency (EMA/CVMP/858971/2022). mRNA vaccines against several types of cancer were also studied in the last two decades [21]. Thus, DNA vaccines will be probably used in the coming years and not only for vaccination but also to selectively introduce genes of biomedical interest [57].

### 3.2. Nanoparticles in Biomedicine

Nanoparticles (NPs) showed unique physico-chemical features for use in biomedicine. Most of the available nanomedicines, both in the clinic and the research field, such as Doxil, Abraxane, or Onpattro, demonstrated promising performances against several serious and complexes illnesses, such as cancer or autoimmune diseases [58]. In this sense, nanotechnology is expected to impact in virtually all fields of current medicine, from diagnosis to disease monitoring, going through surgery and chemotherapy or regenerative medicine. However, despite their great potential, there are still no more than a hundred nanomedicines on the market [59,60], which, depending on their composition, can be divided into five main groups [61,62]: (1) paramagnetic iron oxide nanoparticles, which are mainly used as contrast agents for magnetic resonance imaging techniques in order to obtain information on the location and dimensions of possible tumors [63]; (2) liposomes and lipid nanoparticles, which are nanoparticles composed of phospholipids, the same material that cell membranes are made of, and, therefore, have excellent biocompatibility properties [64]; (3) therapeutic polymers, which include nanoparticles made of biodegradable polymers, such as PLGA nanoparticles with different encapsulated drugs [65]. Drug release will occur as the nanoparticle biodegrades in the patient’s body; (4) protein nanoparticles, in which certain proteins, such as albumin, are used to encapsulate cytotoxic drugs [66]; and (5) drug-antibody conjugates, which constitute a category of their own and in which antibodies guide drugs to specific target tissues [67].

Among the potential applications of NPs, their recent use in vaccine technology expanded the possibilities offered by conventional vaccines [68]. The different types of drug delivery systems based on nanoparticles can be designed to increase the stability and immunogenicity of antigens. The shape, size, and surface charge of the NPs are determinant in the interaction of these vaccines with antigens and the cells of the immune system, and also condition the target or site of action and the elimination of the NPs. The most important NPs-based drug delivery systems used in vaccines are those capable of providing a potent and antigen-specific immune response. The next section describes the types of NPs most frequently used in vaccine development.

### 3.3. Nanoparticles Employed in Vaccines Technologies

While nanoparticles have great potential to carry and release drugs, they also have the potential to act as antigens themselves [69]. In this sense, nanoparticles could be specifically engineered to interact with the immune system, which could be desirable when leading to certain beneficial biomedical applications, such as vaccine development of certain therapies for inflammatory and autoimmune disorders. This technology makes it possible to add a series of agents that direct the nanoparticle towards a specific target, or that enhance the immune response to the antigen. On the other hand, it was also shown that, in some cases, the nanoparticle itself is able to enhance the immune response induced by the antigen [70]. In fact, the most important NPs-based drug delivery systems used in vaccines are those capable of providing a potent and antigen-specific immune response. 

There are certain parameters that are crucial for nanoparticle immunogenicity, such as size, shape, and surface charge of the nanoparticles themselves [71]. The reason for that relays on their importance to improve antigen delivery and presentation. In this regard, they have a strong influence on NP circulation, biodistribution, bioavailability, and capacity to cross certain biological barriers. 

Nanoparticle size was found to determine the way of cellular uptake, together with the cellular specificity and migration, so they can reach antigen presenting cells to activate the immune response [72]. The particle size can significantly contribute to the efficiency of vaccine formulations, since it was reported as one of the most important factors in determining if the loaded antigens induce type I (interferon-gamma) or type II (IL-4) cytokines, which would ultimately determine the immune response [73,74]. Examples of how nanoparticle size might be a leading parameter to determine the potential to induce cytokine responses are the length of CNTs, which was observed to correlate with the induced subcutaneous inflammation in an in vivo model [75]. 

An important consideration is that the immune system recognizes foreign bodies based on their size, among other properties. Large NPs normally interact with APCs present in many tissues, while NPs smaller than 200 nm could circulate for longer through the venous system and lymphatic drainage, which increases the antigen presentation [76]. Interestingly, nanoparticles with size ca. 50 nm were found to increase the expression of certain cell markers and inflammatory cytokines that are responsible for the immune system [77].

The shape of the NPs also has a strong influence in the cellular interaction, intracellular trafficking, and the release kinetics of the antigen into de-targeted cells. Nanoparticle shape can also determine the localisation of nanoparticles inside the cells, as it happened with nano rods vs. nano sheets. The former was delivered to the nucleus while the latter were retained into the cytoplasm [72]. Therefore, nanoparticle shape would also control the immune response of the NPs. There are reports showing that rod-shaped NPs that might present higher surface area than spherical NPs are more likely to be internalized by macrophages, enhancing the production of certain inflammatory markers [78]. 

The surface charge of the NPs is responsible for the interaction with the molecules of the membranes of the targeted cells. In this sense, positive NPs would be more efficiently internalised by the antigen presenting cells than neural or negatively-surface charged NPs, which might help to generate a stronger immunological response [79]. 

**Figure 5:**
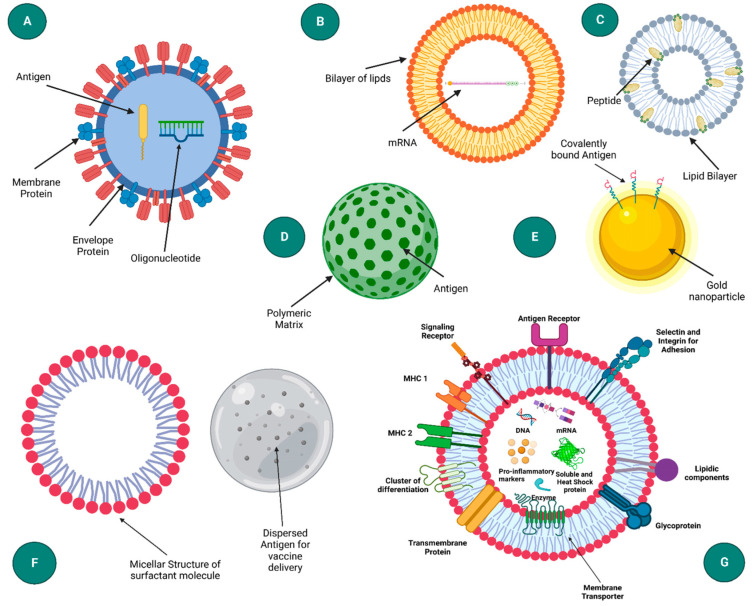
Illustration of different types of nanoparticle delivery structures employed in the development of vaccine technologies. (**A**) virus-like particle, (**B**) liposome, (**C**) immune stimulating complexes, (**D**) polymeric nanoparticle, (**E**) inorganic nanoparticle, (**F**) emulsion and (**G**) exosome. Reproduced from [80].

In this section, a selection of the types of NPs most frequently used in vaccine development is described (Figure 5).

Regarding the type of nanoparticles employed for the development of SARS-CoV-2 vaccines, it was observed that LNPs that present a size of ca. 150 nm could induce a higher immune response than those of ca. 65 nm, which highlights the importance of the correct selection of the size of the nanoparticles for vaccine development [81].

#### 3.3.1. Polymeric Nanoparticles

Polymeric NPs called the attention of the biomedical area over the last few years due to their unique properties and behaviors resulting from their small size and composition [82]. This type of NPs shows a great potential for a wide range of biomedical applications, such as diagnostics and drug delivery. Polymeric NPs attracted much attention in the vaccine world for two main reasons: their ability to release antigens and their biodegradability. They can be prepared from many types of polymers, and their biodegradation kinetics, and, hence, their release kinetics can be tuned and by varying their composition or molecular weights. The most common are NPs prepared from poly(lactic acid) or polymeric mixtures of poly(lactic) and poly(glycolic acids), which are often prepared by different nanoprecipitation techniques [82].

#### 3.3.2. Virus-Like Nanoparticles

As their name suggests, virus-like nanoparticles are composed of a self-assembled layer made of virus capsid proteins, so that a strong immune response can be achieved [83]. In this type of nanoparticles, the antigen can be exposed on both the surface or inside the particles. An important factor of these vaccines is that, despite being composed of a virus protein, they self-assemble without encapsulating viral RNA, so they will not replicate and will not be infectious. In fact, vaccines formulated with virus-like nanoparticles were one of the first class of nanoparticles to reach the market, specifically against hepatitis B virus in 1986, followed by vaccines against human papillomavirus in 2006 and hepatitis E in 2011 [84].

#### 3.3.3. Immune Stimulating Complexes

The immune stimulating complexes (ISCOMs) are vaccine delivery systems consisting of colloidal saponins (glycosides) together with phospholipids and cholesterol [85]. They are structured as geometrical arrangements of micelles containing the saponin and lipids, which are held together by hydrophobic interactions and stabilized through their negative surface charge. Several antigens were investigated with this system; for example, antigens derived from herpes simplex or influenza virus. These lipid-based nanoparticles already showed potential as adjuvants and vectors for certain antigens aiming at prophylactic and/or therapeutic vaccination through different ways of administration [85].

#### 3.3.4. Inorganic Nanoparticles

From an inorganic point of view, the most studied nanoparticles in the area of vaccine technology are gold, carbon, and silica NPs, and most of them focused on transporting different types of DNA plasmids expressing different antigens, such as influenza or hepatitis B [86,87].

#### 3.3.5. Liposomes and Lipid Nanoparticles

One of the most important components of both liposomes and lipid nanoparticles are phospholipids, so this is why they exhibit an excellent biocompatibility. Liposomes have a lipid bilayer on the surface with an aqueous core where they can encapsulate the pathogen antigens, if they are hydrophilic, or in the bilayer, which can be single or multilayered, if they are hydrophobic [88]. However, one of the weaknesses of liposomes is that they are easily degraded, either by the action of certain enzymes, pH, or the immune system, which is why more robust systems such as lipid nanoparticles were developed [87]. 

Unlike conventional liposomes, which use cationic lipids to transport negatively charged nucleic acids, lipid nanoparticles use ionizable cationic lipids, which remain neutral in the bloodstream, thus reducing their potential toxicity. They only become positively charged inside cells, favoring the intracellular release of the transported nucleic acid. In addition, these LNPs also contain cholesterol, to promote their stability and some flow capacity; other conventional phospholipids that help package messenger RNA; and polyethylene glycol on their surface, to promote their stability and protect them from the immune system [86]. Most LNPs compositions are very similar and where there is perhaps more variation, and, of course, more protected intellectual property, is in the ionizable lipids, which is one of the major differences between the vaccines developed by Moderna and Pfizer BioNTech, with all other ingredients being virtually the same. 

Normally, LNPs are produced using a microfluidic system where the aqueous phase containing the RNA is mixed with the organic phase containing the lipids dissolved in ethanol through microchannels, resulting in the precipitation of lipid nanoparticles. The size of the produced LNPs can be controlled with the gradient and speed of the precursor flows when they mixed up to nanoprecipitate the particles [89].

### 3.4. Nanoparticle Platforms as Vaccine Adjuvants

Adjuvants were conventionally described as those substances that in combination with a specific antigen can produce a more robust immune response than the antigen alone. Thus, nanoparticles can deliver either antigens or adjuvants to the targeted cells at predetermined rates and durations looking for optimal immune responses. Taking into account that nanoparticles can encapsulate antigens preventing their premature degradation and prolonging the antigen exposure, they can act as delivery platforms and adjuvants simultaneously. In fact, the different composition, size and morphology of nanoparticles allow them to selectively induce many different types of immune responses and/or release the transported antigens into specific sites, as it was commented above. Some typical adjuvants employed in vaccine technologies included both inorganic nanoparticles, such as aluminum, calcium phosphate, gold, and silica nanoparticles; and organic nanoparticles, such as chitosan or lipid-based nanoparticles [90]. 

### 3.5. Interaction with the Body

The SARS-CoV-2 virus has a series of proteins on its surface, the spike protein, which resembles a corona; hence, this type of virus is called corona virus. It is through this spike protein that the virus enters our body, binding to a membrane receptor, ACE2, present in the epithelial cells of the lungs [91]. In this way, the cause of the contagion, the spike protein, is on the surface of the virus, so if we are able to teach the immune system that these proteins represent a threat, it would be very easy to recognize [19]. Additionally, it was for this reason that the lipid nanoparticles in the RNA vaccines carry a fragment of messenger RNA that codes for the spike protein. The nanoparticles carry the messenger RNA and introduce it inside certain cells, where the spike protein will be produced thanks to the information we introduced and, thus, be able to produce the antigen. Thus, the cell itself that produced them knows that it is an exogenous antigen and tries to send it out of the cell so that the immune system comes and recognizes it. From there, the immune system can respond in various ways [91]. One of them is through the B cells, which have specific antibodies for this type of protein, and when it binds to the antigen, it is able to produce antibodies against that antigen. In this way, in the event that in the future some type of SARS-CoV-2 virus enters our body, our immune system will be prepared, with antibodies ready to bind to the spike protein of the virus. When it completely surrounds it, this marks the virus for elimination in a way that neutralizes it. That is, the vaccine taught our B cells to produce antibodies that will then neutralize the actual virus even before it infects any cells. 

**Figure 6:**
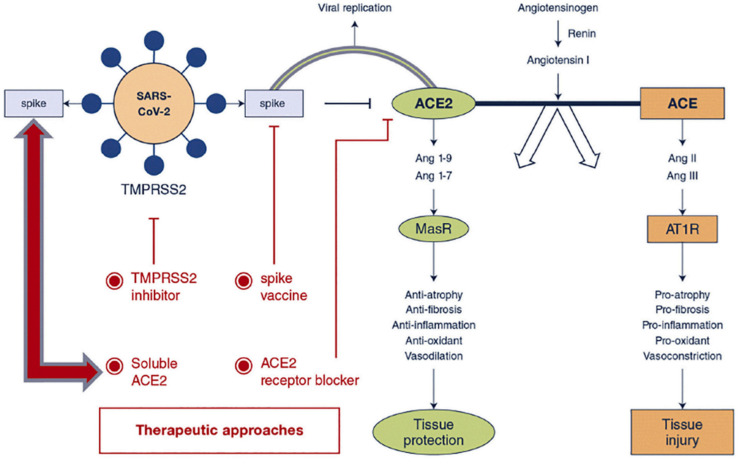
Effect of Angiotensin-converting enzyme-2 in the pathophysiology of COVID-19 and the subsequent receptor blockade for the treatment of the disease. Reprinted with permission from [91] 2020, Elsevier B.V.

The other form of response by our immune system is based on the involvement of B cells in another cell signaling process by communicating with a T cell that launches the cell signaling process and communicates with an antigen presenting cell, which, in turn, can communicate with another type of T cell that can kill other cells that were infected by the virus [92]. Then, in case there is any cell infected by the virus, it will be recognized by the cytotoxic T cells that will kill that infected cell. Therefore, there are a couple of different responses, but the important thing is that the B cells will either produce antibodies to fight the virus or initiate cell signaling sequences to kill infected cells (Figure 6) [91,92,93].

## 4. Success Case of Vaccines Based on Nanoparticles to Fight SARS-CoV-2

Nanoparticles constitute promising delivery vectors for effective and safe vaccines. Among them, biologically derived nanoparticles, such as virus-like particles, extracellular vesicles, and protein nanocages, are able to mimic both the structure and function of live pathogens, being unable to replicate and, therefore, are not infectious. While a comprehensive review on this type of vaccines can be found somewhere else [94], we will focus the next section on the success of nanovaccines for the treatment of SARS-CoV-2. 

The SARS-CoV-2 pandemic saw an extraordinary effort on vaccine development which resulted in the extraordinary success of nucleic acid vaccines, mainly mRNA vaccines. The use of these vaccines confirmed their usefulness against intracellular pathogens, such as viruses with a high mutation rate, bacteria including *Mycobacterium*, or parasite protozoa which evade host antibodies by infecting and residing in host phagocytic cells. In these cases, the cellular immune response, especially that of CD8^+^ cells, resulted in better and longer protection against these types of pathogens given that protection profits from durability of cellular action as well as from extent of memory capability, which contributes to contain reinfections [95]. 

The SARS-CoV-2 pandemic is still a primary concern worldwide despite the success of vaccines that protected the vaccinated population from severe symptoms. Most are effective at mitigating the severity of the disease [96] and are mostly based on the Spike (S) antigen of the SARS-CoV-2 virus surface delivered in adenoviral vectors encoding the protective antigen genes either as mRNA molecules or DNA plasmids, and the more classic recombinant protein formulation. One of the most successful types was mRNA molecules included in lipid nanoparticles which induced high protection levels [97,98,99,100,101]. However, these vaccines require storage temperatures between −20 °C and −80 °C for distribution to avoid mRNA degradation, which is a serious drawback for developing countries. Additionally, current dominant variants, such as Omicron (B.1.1.529) and other variants of concern (VOCs), are not very sensitive to these vaccines [102]. Adverse effects, such as anaphylaxis and myocarditis, are rare (reviewed in [96]). Additional vaccination does not tackle vaccine escape variants [103].

Non-replicating viral vector vaccines also require cold chain distribution (2–8 °C) and cause rare adverse events, such as thrombosis with thrombocytopenia syndrome, Guillain–Barré syndrome, and proinflammatory response [96,104]. Second generation vaccines, which are based on recombinant proteins formulated with adjuvants, lead to high specific antibody responses but low T-cell activation levels [20,27]. T-cell responses do not fade as quickly as antibody responses and are more effective at protecting against emerging variants [105,106,107,108]. Vaccines protecting against a broader spectrum of VOCs and eliciting a robust and lasting cellular response will be necessary to limit the impact of new VOCs [109].

DNA vaccines, which were used solely in veterinary medicine until recently (see above), were shown to be effective at protecting humans [93] and the mouse model [52] from SARS-CoV-2. These vaccines are easily modifiable to protect from new virus strains; they are thermotolerant, and the cold chain is not required for long-term storage or worldwide distribution, which would benefit low-income countries [93,110]. Several vaccine candidates were tested during the pandemic [93]. It is interesting to note the fact that T-cell responses seem to play an important role in SARS-CoV-2 infection. Strong T-cell responses correlated with recovery of patients who had suffered mild disease [111,112]. A robust CD8^+^ T-cell response with broad specificity is considered a sign of successful protective immunity against SARS-CoV-2 [113]. Patients who tested positive for CD4^+^ and CD8^+^ T cells are less sensitive to reinfection [95], highlighting the need for vaccines that elicit a potent cellular immune response. DNA and mRNA vaccines are able to stimulate a robust T-cell immune response.

There is a DNA vaccine against SARS-CoV-2 containing the N protein from the viral nucleocapsid as protective antigen in addition to the S antigen which was shown to be fully protective in the mouse model [52]. In some viral infections, non-neutralizing antibodies directed to the nucleoprotein can help to clear the infection of enveloped viruses [114,115,116]. It seems that the E3 ubiquitin ligase TRIM21 can use anti-N antibodies to target the N protein proteasomal degradation, which triggers the activation of effective cytotoxic T-cell responses against the N antigen [117]. Thus, the inclusion of the N protein antigen in SARS-CoV-2 vaccines improves protection, as was demonstrated in the K18-hACE2 mouse model for COVID-19 disease [52]. The addition of other pathogen antigens may improve the quality and intensity of the protective immune response. In the case of SARS-CoV-2, a vaccine encoding two or more protective antigens may help prevent the spread of infection in target organs, resulting in improved recovery and a decrease in the long-term clinical signs detected in the disease [52].

On the whole, nucleic acid vaccines are an excellent tool for the prevention of infections caused by viruses and intracellular pathogens. The hurdle to overcome is the accessibility of APCs to specific pathogen antigens produced by cells of the vaccinated host. Nanoparticles composed of lipids or other materials seem to be an excellent vehicle to help achieve this.

### 4.1. Jonhson & Johnson/Jansen and Oxford/Astra Zeneca Vaccines

The development of these vaccines was based on using a known DNA technology, where an adenovirus vector was engineered to carry the DNA with the information to produce the surface spike protein of the COVID virus. Concretely, the Johnson & Johnson vaccine is based on an adenovirus type 26 modified to produce the SARS-CoV-2 Spike protein. This adenovirus vaccine was designed to be employed as a single intramuscular injection—the first of this type available that comes in a single dose—and when it enters a cell, it produces the vaccine protein but cannot replicate inside the cell or cause illness. This vaccine was shown an average of 66% protection against moderate or severe COVID-19, but more importantly, this vaccine showed 85% protection against severe disease, with no differences across countries or age groups. 

The Oxford/Astra Zeneca vaccine is also based on DNA transported by an adenovirus vector—a modified version of a chimpanzee adenovirus, known as ChAdOx1—to transport the genetic information to produce the surface spike protein of the virus. One of the benefits of this type of vaccines is that they are more rugged than the mRNA vaccines. This is because DNA is not as fragile as RNA, and the adenovirus’s tough protein coat helps protect the genetic material inside. Consequently, the Oxford/Astra Zeneca vaccine does not have to be stored frozen. In fact, this vaccine was expected to last for at least six months when refrigerated at ca. 4 °C. In 2020, it was shown that the efficacy of the vaccine was ca. 76% at preventing COVID-19 following the first dose and ca. 81% after the second dose [118].

### 4.2. Moderna and Pfizer Vaccines

The development of SARS-CoV-2 vaccines based on the use of messenger RNA technology encapsulated in lipid nanoparticles was a success case in the use of nanoparticles for vaccine manufacture [44]. Messenger RNA is the key intermediary in protein synthesis, but it is a very large and negatively charged molecule, so it cannot cross cell membranes on its own. Thus, messenger RNA needs a vehicle to cross these cellular barriers, and this is where LNPs played a major role. 

While it is true that over the past few years, we had the misfortune of experiencing a pandemic first-hand, it is also true that we were fortunate to witness in real time how we came out of this pandemic thanks, among other things, to the extraordinarily rapid development of SARS-CoV-2 vaccines [119]. In the case of Moderna’s vaccine, development was truly rapid; from 11 January 2020, when Chinese scientists published the genetic sequence of the virus, it took just a couple of days for materials scientists to design a messenger RNA vaccine, encapsulate it in nanoparticles, and send it to the NIH in February to begin trials. The first patient was injected with the first prototype vaccine on 16 March 2020, the start of human clinical trials. Two months later, in May 2020, Phase I results were published, and then, 6 months later, Phase II and III results were presented, with 94–95% effectiveness (as a reference, the flu vaccine should be around 45% effective), in what was a giant victory for science and for nanoparticle technology [100,101].

On the other hand, Pfizer signed a letter of intent with BioNTech to co-develop a potential COVID-19 vaccine just six days later than the World Health Organization declared the pandemic on 11 March 2020. By that time, BioNTech, a German immunotherapy company, pioneered a novel genetic technology based on mRNA to prompt cells to fabricate antibodies to fight off that virus. Although that mRNA technology was out there for decades, problems related to the structure and stability of mRNA prevented its translation to the clinic. In this sense, BioNTech had a great success stabilizing mRNA thanks to the use of lipid nanoparticles. Over the next nine months, Pfizer and BioNTech worked together to develop a vaccine across companies and across countries to set a record in any previous vaccine development program. 

All this was possible thanks to the research work of many people over a long period of time, both from the point of view of messenger RNA technology and from the point of view of nanoparticle technology. Many people were involved in the whole process of generating the knowledge and basic science necessary for its application in a specific case such as this. Among them, it is worth mentioning the pair of Turkish doctors who founded BioNTech, Tureci and Sahin, the great messenger RNA specialist, Kariko, the founders of Moderna, Rossi and Langer, and, of course, Pieter Cullis, a pioneer in the use of lipid nanoparticles to transport messenger RNA into cells [120,121].

## 5. Conclusions and Final Remarks

Over the past few years, various nanosystems were developed as vaccine carriers, each of which has certain advantages and/or disadvantages over existing vaccine delivery approaches. Throughout this review, it was demonstrated that the use of NPs to deliver vaccine components can be advantageous for the treatment of infectious diseases. The success of this technology lies in the fact that nanoparticles can easily encapsulate a large number of active biomolecules, such as antigens, proteins, or targeted nucleic acids, protecting them from degradation. One of the advantages of these systems is that nanoparticles can provide a specific release of cargo into draining lymph nodes after crossing biological barriers, thus producing long-lasting immunological effects. As this review showed, certain nanoparticles can be quite effective in eliciting both cellular and humoral immune responses that would not otherwise be possible with traditional vaccines. The recent success of nanoparticle-based SARS-CoV-2 vaccines generated a lot of confidence in the medical community about nanovaccines. In fact, the NP-based vaccine delivery strategy is gaining great potential as a delivery platform for human infectious diseases and it is expected that more nanovaccines will be on the market in the near future.

## Data Availability

Not applicable.
